# Convergent evidences from human and animal studies implicate angiotensin I-converting enzyme activity in cognitive performance in schizophrenia

**DOI:** 10.1038/tp.2015.181

**Published:** 2015-12-08

**Authors:** A Gadelha, A M Vendramini, C M Yonamine, M Nering, A Berberian, M A Suiama, V Oliveira, M T Lima-Landman, G Breen, R A Bressan, V Abílio, M A F Hayashi

**Affiliations:** 1 Integrated Laboratory of Clinical Neurosciences and Schizophrenia Program, Departamento de Psiquiatria, Universidade Federal de São Paulo, São Paulo, Brazil; 2 Departamento de Farmacologia, Universidade Federal de São Paulo, São Paulo, Brazil; 3 Departamento de Biofísica, Universidade Federal de São Paulo, São Paulo, Brazil; 4 Medical Research Council Social, Genetic and Developmental Psychiatry Centre, Institute of Psychiatry, King's College London, London, UK

## Abstract

In schizophrenia (SCZ), higher angiotensin I-converting enzyme (ACE) levels have been reported in patient's blood and cerebrospinal fluid (CSF). Hereby, we propose to explore whether the ACE activity levels are associated to cognitive performance in SCZ. Seventy-two patients with SCZ or schizoaffective disorder diagnosis, and 69 healthy controls (HCs) underwent a cognitive battery with parallel collection of peripheral blood samples to measure ACE activity. Significant higher ACE activity levels were confirmed in the plasma of SCZ patients compared with HCs (Student's *t*=−5.216; *P*<0.001). ACE activity significantly correlated to Hopkins delayed recall measures (*r*=−0.247; *P*=0.004) and Hopkins total (*r*=−0.214; *P*=0.012). Subjects grouped as high ACE activity (above average) had worse performance compared with low ACE activity level group for Hopkins delayed recall measure, even after correction for clinical condition, age, gender and years of education (*P*=0.029). The adjusted *R* squared for this final model was 0.343. This result was evident only comparing extreme groups for ACE activity, when splitting the sample in three groups with similar number of subjects. To clarify this finding, we performed an evaluation of the cognitive performance of transgenic mice with three copies of ACE gene in novel object recognition (NOR) test, which showed that such animals presented impairment in NOR (*P*<0.05) compared with two copies of wild-type animals. The results observed in SCZ patients and animal model suggest both the association of ACE to cognitive deficits in SCZ. This finding may support the evaluation of novel treatment protocols and/or of innovative drugs for specific intervention of cognitive deficits in SCZ envisioning concomitant ACE activity and behavior evaluations.

## Introduction

The renin–angiotensin system (RAS) is viewed as an important regulator of blood pressure and body fluid balance, although many other functions in the central nervous system have been related to RAS, including several associations with psychiatric diseases and cognitive functions.^1, 2, 3, 4, 5, 6^ Several lines of evidence support a role for RAS in schizophrenia (SCZ). First, the regulatory effects of RAS on brain dopamine pathways.^7, 8^ Second, the specific activation of the angiotensin type 2 receptor in neuronal cells is able to induce neurite outgrowth and elongation, to modulate neuronal excitability and cellular migration, affecting therefore, the synapse plasticity and neurodevelopment, as reviewed.^9^ Third, the key component of RAS, that is, the angiotensin I-converting enzyme (ACE), although primarily studied owing to its ability to convert angiotensin I (Ang I) into the hypertensive peptide angiotensin II (Ang II),^10^ is also capable to cleave peptides as neurotensin and substance P, which were associated with SCZ, as independently reported by different groups.^11, 12, 13^

The first study of ACE in SCZ reported a reduction in the enzyme activity in the basal ganglia of postmortem brain of SCZ patients compared with healthy controls (HCs) that was not replicated later.^14, 15^ Also in studies of cerebrospinal fluid (CSF) of patients, paradoxical findings can be found, as both reductions and increases in ACE activity levels in CSF of SCZ patients were described.^16, 17^ Wahlbeck and colleagues^18, 19^ also suggested that ACE activity levels in CSF may be correlated to the length of illness and to the use of antipsychotics. Finally, using a smaller sample, Baskan *et al.*^20^ reported a significant increase in ACE protein level in the serum of SCZ patients compared with HCs.

The ACE gene, located on chromosome 17q23, has a well-studied insertion/deletion polymorphism resulting from the presence or absence of a 287-base pairs fragment in the 16th intron of ACE gene, in which the presence of D-allele is associated with a higher ACE activity in serum.^21, 22^ In a recent report, we showed that ACE activity levels in plasma were higher in patients with SCZ and the differences between the observed ACE activity and the expected mean values of each genotype groups were the best predictor for the clinical status (that is, patient vs HCs).^23^

Interestingly, higher ACE activity levels were also previously reported in Alzheimer's and Parkinson's diseases, and several lines of evidence suggested a role for RAS in cognitive functions, especially memory.^5, 24, 25^ Ang II administration seems to interfere with cognitive performance in memory tests in animal models, whereas ACE inhibitors and angiotensin type 1 receptor antagonists improve cognitive processing in both humans and animal models.^24, 25, 26, 27^ There are several evidences for the Ang II action in the central nervous system associated to cognitive functions, more specifically to memory processing.^28^ However, the role of Ang II is still controversial, with some studies suggesting an amnesic effect,^29, 30, 31^ whereas others propose that Ang II is able to improve memory.^32, 33^

Cognitive deficits are a central feature of SCZ and major determinants of functional impairments, with more than 75% of patients depicting significant cognitive impairment.^34, 35^ Several domains are affected, but the effects on executive function and memory are usually the most prominent.^36^

Therefore, in this study, we aim to clarify whether ACE activity levels in the plasma of SCZ patients and HCs are associated to cognitive performance in a neuropsychological battery. In addition, we also conducted behavioral evaluations of cognitive function of transgenic mice carrying three copies of the ACE gene,^37^ aiming to verify the influence of higher expression levels of the ACE in cognitive processing, and ultimately aspiring the comparison of the data from human and animal models.

## Materials and methods

### Study in patients with SCZ

#### Study participants

In this study, 72 patients with SCZ or schizoaffective disorder diagnosis and 69 HCs, aged from 18 to 65 years fulfilling the inclusion and exclusion criteria and that completed the neurocognitive battery, were enrolled. All patients were enrolled in the SCZ Community Treatment Program (PROESQ) run by the Universidade Federal de São Paulo (UNIFESP) for at least 1 year. The inclusion criteria were the diagnosis for SCZ or schizoaffective disorder according to DSM-IV. This sample is part of a larger sample enrolled to multimodal research on SCZ. All the subjects that fulfilled inclusion and exclusion criteria for the current analyses were included.

HC volunteers were selected, matching by age, gender and educational level, from a governmental unemployment agency. Controls were first screened by telephone for psychiatric diagnosis and then invited to a face-to-face, full psychiatric interview. Inclusion criteria were no current or lifetime psychiatric diagnosis, and absence of family history of psychosis in first- and second-degree relatives.

Exclusion criteria for both groups were a diagnosis of arterial hypertension, use of any antihypertensive medication, for example, propranolol or lack of consensus diagnosis.

The Structured Clinical Interview for DSM-IV was performed for both patients and HCs by trained psychiatrists to assess the psychiatric diagnosis. A questionnaire adapted from the Structured Clinical Interview for DSM-IV screening questions for each diagnosis was used to investigate the family history of mental disease in first and second degree, information was collected by patient and accompanying relative. The clinical assessment for patients also included the Positive and Negative Syndrome Scale and Global Assessment of Functioning. For the diagnosis, we used all the available information including medical records.

This study was previously approved by the Research Ethics Committee of UNIFESP (CEP No. 1883/10). Written informed consent was obtained from all the participants recruited and clinical and laboratory investigations were strictly conducted according to the principles expressed in the Helsinki Declaration.

Neuropsychological tests: Eight tests, assessing different aspects of executive functions, working memory and verbal memory, were administered. Intelligence quotient (IQ) was also measured for all the subjects. In a previous study, the differences observed in the performance of these tasks allowed a good discrimination between subjects with SCZ and HCs.^38^ Assessments were not blinded for case–control status. Well-established and widely used neuropsychological tests used in SCZ research were used here and they are listed and shortly described below:

Working memory/updating—Letter memory task (adapted from Morris and Jones^39^)—single letters were presented serially to the participants who were instructed to create a new string of letters by mentally adding the most recently presented letter, dropping the third letter back, and then reciting the new string of two letters aloud. This instruction was given and doubled-checked for understanding to guarantee the participants' continuous updating.

—Keep track task (adapted from Yntema^40^)—initially, several target categories were shown to the participants on the computer screen. Fifteen words, including two or three examples from each of the target categories (animals, colors, countries, distances, metals and relatives) were verbally presented in both serial and random orders, while the target categories remain at the bottom of the computer screen. The subject was first asked to remember the last word presented in each of the target categories, and then to say his/her answers aloud at the end of the trial.

Inhibition—Victoria Computerized Stroop Test^41^—the test was composed of three parts, each comprising 24 stimuli. In the first part, the subject was asked to read the word that appears in the computer display and the stimuli were the name of one of four chosen colors (that is, yellow, blue, green or red) written in capital black font. In the second part, colored circles were displayed in the screen for 40 ms, and the participants have to name the color of the circle as fast as they can, aiming to provide a baseline measure for the analysis of errors and reaction times. In the third part, the subjects were asked to read the color name, but the stimuli were divergent, that is, the word was displayed for 40 ms in a color different from the color names. The scores for reaction time and for the number of correct answers were based on the subtraction between the mean results obtained in the third and second parts of this test.

Set-shifting tasks—trail making test^42^—participants were asked to complete two tasks, one requiring to draw lines to connect circled numbers in a numerical sequence, and the other requiring to connect circled letters in alphabetical order. The outcome variable was the mean of the total time taken to complete the two conditions minus the total time taken to complete the last phase of the task.

—Plus–minus task (adapted from Jersild^43^)—three lists of 30 two-digit numbers were presented. On the first, participants need to add 3 to each number. On the second, they have to subtract 3. On the third, they were required to alternate between adding and subtracting 3. The outcome was the mean of the total time taken to complete the two first phases minus the total time taken to complete the third phase.

—Letter–number task (adapted from Rogers and Monsell^44^)—a letter–number pair is presented in one of four quadrants on the computer screen. Subjects have to indicate whether the number was odd or even, when the pair was presented in any of the top two quadrants. Then, they have to indicate whether the letter presented in the bottom two quadrants was a consonant or a vowel. Finally, stimuli were presented in all the four quadrants, and the participants have to shift between these two types of operations. The outcome measure was the mean of the total time taken to complete two first phases minus the total time taken to complete the last phase.

Verbal learning and memory—Hopkins verbal learning task revised (HVLT-R)^45^ or Hopkins delayed recall—participants were requested to read the same list of words three times, and each time they are asked to repeat as many words as they could recall. In this study, we used only the word-list learning score. After 30 min, they were asked to recall the list again; allowing the delayed recall or evocation measure.

Complex executive functions—The Wisconsin card sorting task with 64 trials^46^—participants were requested to match cards presented with any one of four reference cards shown on a table. They were instructed to sort the cards into piles under the reference cards, according to categories that must be perceived by them. Response cards could be matched on color, shape or number. Once the participant made 10 consecutive correct sorts, the sorting principle changed. The score of interest was the number of the perseverative errors.

Non-verbal intelligence test—The non-verbal intelligence task (R-1)^47^ was developed to allow measurement of intelligence in low-literacy populations. This test is highly correlated with the Raven's progressive matrices test (*r*=0.76, *P*=0.001) and was chosen owing to the high frequency of illiteracy found in the population of Brazilians with SCZ.

#### Direction of results

For Hopkins measures, higher values indicate better verbal learning and memory retrieval. For all the other tests, higher values indicate worse performance.

#### Blood samples

Blood samples were collected from all the subjects into sodium heparin vacuum tubes BD Vacutainer (BD Medical—Pharmaceutical Systems, Franklin Lakes, NJ, USA). The samples were then centrifuged at 1500–2000 *g* for 10–15 min, at room temperature, to recover the plasma, which was then stored at −20 °C in microcentrifuge plastic tubes (Axygen, Union City, CA, USA) until use. The plasma was carefully removed with a transfer pipette without disturbing the white blood cells layer. Although the suggested procedure is to fractionate the blood as soon as possible after collection, some samples were kept at 4 °C up to 24 h after blood collection and then centrifuged for plasma recovery, with no detectable influence in the measured enzymatic activity (data not shown). The stored plasma samples were defrosted in wet ice soon before the activity measurements as follows.

#### Activity measurements

The ACE activity in human plasma samples of HC volunteers and SCZ patients were measured by fluorometry, using the FRET peptide substrate Abz-FRK(Dnp)P-OH.^48^ The researcher responsible for the measures was masked for the group of the sample for humans and animals. Hydrolysis of the substrate (10 μm), at 37 °C, was monitored by measuring the fluorescence in a Shimadzu F-7000 spectrofluorometer at *λ*_em_=420 nm and *λ*_ex_=320 nm. A 96-wells plate containing 100 μl of substrate solution (50 mm Tris-HCl pH 7.4 and 100 mm NaCl buffer) in each well was placed in a thermostatically controlled cell compartment for 5 min, before the addition of the plasma samples. The increase in fluorescence (arbitrary fluorescence units) with time was continuously recorded for 5–10 min, both in the absence and in the presence of the ACE inhibitor lisinopril (Sigma Aldrich, St. Louis, MO, USA). The measured ACE activity is the rate of hydrolysis in the absence of the specific inhibitor minus the rate determined in the presence of the inhibitor. The average ACE activity in the human plasma was determined by averaging the ACE activities of three (triplicate) measurements for each sample, and then if these values variations were higher than 5% from each other, new measurements (at least two more measures, for example, duplicates) were performed to assure that the final average for all samples used in the experiment were not because of any technical influence.

### Animal study

#### Animals

To investigate the role of ACE, adult male C57BL/6J mice genetically engineered carrying a duplication of the ACE locus on chromosome 11 were used. These mice carry three functional ACE gene copies (+/++ or three copies). Control animals consisted of wild-type C57BL/6J mice (+/+ or two copies). Identification of genetically modified offspring was performed at 21 days of age, by PCR amplification of DNA isolated from ear biopsies. All the animals were housed under conditions of controlled temperature (20–23 °C) and lighting (12 h light/12 h dark cycle, lights on at 0700 h). Food and water were available *ad libitum* throughout the experiments. No randomization was used. All the experiments were performed in accordance with the Guide for the Care and Use of Laboratory Animals of the USA National Institutes of Health (Bethesda, MD, USA). All the experiments with animals were approved by the ethical committee of the Universidade Federal de São Paulo (UNIFESP), CEP No. 0336/12.

#### Behavioral tests

Aiming to provide convergent evidences from both human patients and animal model studies for the association between ACE activity and cognitive deficits, we elected to the novel object recognition (NOR) test,^49^ which is a widely used model for the investigation of short- and long-term memory performance.

This test is based on the spontaneous tendency of rodents to spend more time exploring a novel object than a familiar one.^50, 51, 52^ The choice to explore the novel object reflects the use of learning and memory recognition. During habituation, the animals are allowed to explore an empty arena. Twenty-four hours after habituation, the animals are exposed for 5 min to the familiar arena with two identical objects (A + A) placed at an equal distance. After 1 h, the animal is returned to the apparatus, which now contains the familiar object and a novel object (A + B) to test short-term recognition memory. The next day, the mice are allowed to explore the open field in the presence of the familiar object and a novel object (A + C) to test long-term recognition memory. Animals were recorded by a video camera placed above the apparatus and the time spent exploring each object was registered using the animal video-tracking software Anymaze (Stoelting, Wood Dale, IL, USA). The researchers were blind to the genetic background of the animals.

Randomization was not performed as the animals with different genetic background were not further divided into groups submitted to any additional intervention.

Analysis of the data: For measuring the mean differences between SCZ patients and HC groups, a two-independent-samples *t*-test was used. No significant difference on variance was found for the following measures between groups: ACE activity, Hopkins total, Hopkins delayed recall, keep track test. Significant differences on variance were observed for the other cognitive tasks (data not shown). For this reason, we chose nonparametric correlations (Spearman's coefficient) to investigate the possible associations of ACE activity and cognitive tests.

The whole sample was grouped on the basis of the ACE activity levels in three groups with similar number of subjects as: high (13.7±2.0 nm min^−1^), intermediate (9.9±0.8 nm min^−1^) and low ACE (5.4±2.0 nm min^−1^). We performed a General Linear Model to compare the high and low groups, for age, sex and IQ as covariates. The choice for three groups was to allow the comparisons between the extreme groups.^53^ One advantage for comparing extreme groups is that the overlap between the measures can be reduced, and this would be inevitable if one uses two samples divided by the whole sample median. Aiming to avoid that the result represents solely an effect of distribution of case and control in the groups, clinical status was used as covariate.

In all the adjusted models, we first included age, sex and IQ as covariates, and then, the clinical condition was included to clarify whether the effects could be better explained by the ACE activity levels or by case/control-associated differences.

For the animal model results, normal distribution was confirmed by the Kolmogorov–Smirnov test and the homogeneity of variance was revealed by the Levene's test. A repeated-measures analysis of variance was performed for the NOR (within factor = object 1 and 2, and between factor = genetic background) followed by paired-sample *t*-test. The sample size estimation considered the following parameters: under an analysis of variance (repeated measures, within–between interaction (F-distribution)), alpha error probability, power (1−β error probability), total sample size of 24 allocated in two different groups (*N*=12), correlation among repeated measures at least equal to 0.5 and nonsphericity correction equal to 1 result to a minimum detectable effect size of 0.299. Such effect size is considered by Cohen^54^ as moderate.

The *P*<0.05 was used as a criterion for statistical significance. Sidak procedure was adopted to estimate the corrected *P*-value for multiple comparisons.

Data analyses were performed with the Statistical Package for Social Science (SPSS) Version 20.0 (SPSS, IBM, www.ibm.com/software/analytics/spss/).

## Results

### Human study

#### Subjects' description

The mean age of the whole sample was 33.7 years (s.d.=9.3). No differences were observed for sex, age or educational level between SCZ patients and HC groups (Table 1). Mean duration of illness for patients was 12.4 years (s.d.=7.1).

#### Enzymatic activity of SCZ patients and HCs

ACE activity mean value was significantly higher for SCZ patients (11.0±3.4 nm min^−1^) compared with HCs (7.9±3.7 nm min^−1^; Student's *t*=−5.216; *P*<0.001). No significant correlation was observed between enzymatic levels and total Positive and Negative Syndrome Scale (Spearman's rho =−0.92; *P*=453) or global functioning (Spearman's rho =0.31; *P*=801). No correlations in enzymatic levels were observed in the whole sample with age (Spearman's rho =0.057; *P*=0.504), gender (*t*=1.87; *P*=0.064), years of education (Spearman's rho =−0.056; *P*=0.509). No significant difference on ACE activity level was observed for smoking or number of cigarettes per day (Chi-squared =4.9; *P*=0.179).

Cognitive performance differences between SCZ patients and HCs: Patients had nonsignificant lower IQ mean level. Most cognitive measures showed significant better results for subjects from the HC group, with the exception for the letter memory test (which allow evaluating the updating; Table 2). We checked in the patients' group the effect of antipsychotics using dose equivalents for chlorpromazine. The only significant association was found for the letter memory test (Spearman's rho =0.286; *P*=0.015).

**Figure 1:**
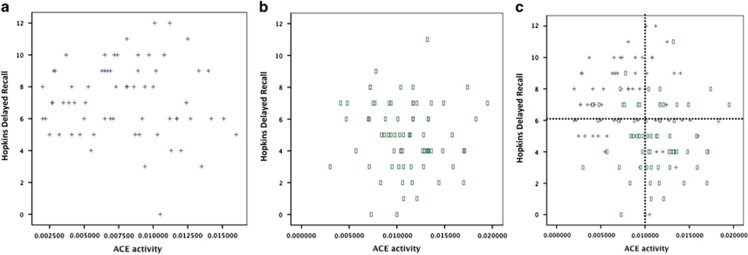
Correlations for memory (evaluated by Hopkins measures) and ACE activity in schizophrenia (SCZ) patients (**a**) and health controls (HCs) group (**b**). The Hopkins plot with patients and HCs together allow to observe a clear trend for lower Hopkins delayed recall and higher ACE activity in SCZ patients compared with HCs (**c**). ACE, angiotensin I-converting enzyme.

Cognitive performance and ACE activity association: Aiming to determine the influence of the measured enzymatic activity on the cognitive performance, the statistical analysis was performed for both the whole sample and for the separated groups. Considering the whole sample, significant correlations for memory (Hopkins delayed recall; *P*_corr_=−0.25; *P*=0.003), verbal learning (Hopkins total; *P*_corr_=−0.219; *P*=0.010) and inhibition (Stroop; *P*_corr_=174; *P*=0.042) correcting for age, gender and IQ were found. Only Hopkins delayed recall remained significant considering *P*-value corrected for multiple testing (Sidak method; *P*-value threshold 0.006). After the addition of clinical condition (patient or HCs) as covariate, the result was no longer significant (*r*=−0.76; *P*=0.377) (results are shown in Table 3). When performing correlations for each group separately, we did not find any significant result (data not shown). The scatter plots of Hopkins delayed recall and ACE activity clearly show marked differences in the pattern of distribution between SCZ patients and HCs, which may explain the results for correlations considering the whole sample and each group separately (Figure 1).

Therefore, the whole sample was initially divided in two groups using the median ACE activity. Then we performed a general linear model comparison between the extreme groups (high vs low ACE activity groups) for each cognitive test using IQ, gender and age as covariates. Significant differences were observed for Hopkins total (*Z*=4.836; *P*=0.030); Hopkins delayed recall (*Z*=9.870; *P*=0.002) and Stroop (*Z*=10.301; *P*=0.002). After adding clinical condition as a covariate, none of these results reached significance considering the *P*-value threshold corrected for multiple comparisons, although Stroop remained significant (*Z*=4.493; *P*=0.036). Then, we split the sample in three groups composed by similar number of subjects as high, medium and low ACE activity categories. In this set of analyses, only Hopkins delayed recall remained significant (*P*=0.029), although a trend was observed for inhibition (Stroop; *P*=0.084). The adjusted *R* squared for the final model with Hopkins delayed recall was 0.343. This final result would not be considered significant considering the threshold for multiple comparisons (*P*=0.006). Therefore, at this point, aiming to better understand our preliminary data in humans, we looked for data on an ongoing animal study validation. We additionally checked the potential effect of antipsychotics on the result and found no significant difference on antipsychotic's dose equivalence for ACE groups, dichotomized (*t*=0.112; df=70; *P*=0.911) or split in three groups (*Z*=1.72; df=2; *P*=0.187).

#### Animal study

**Figure 2:**
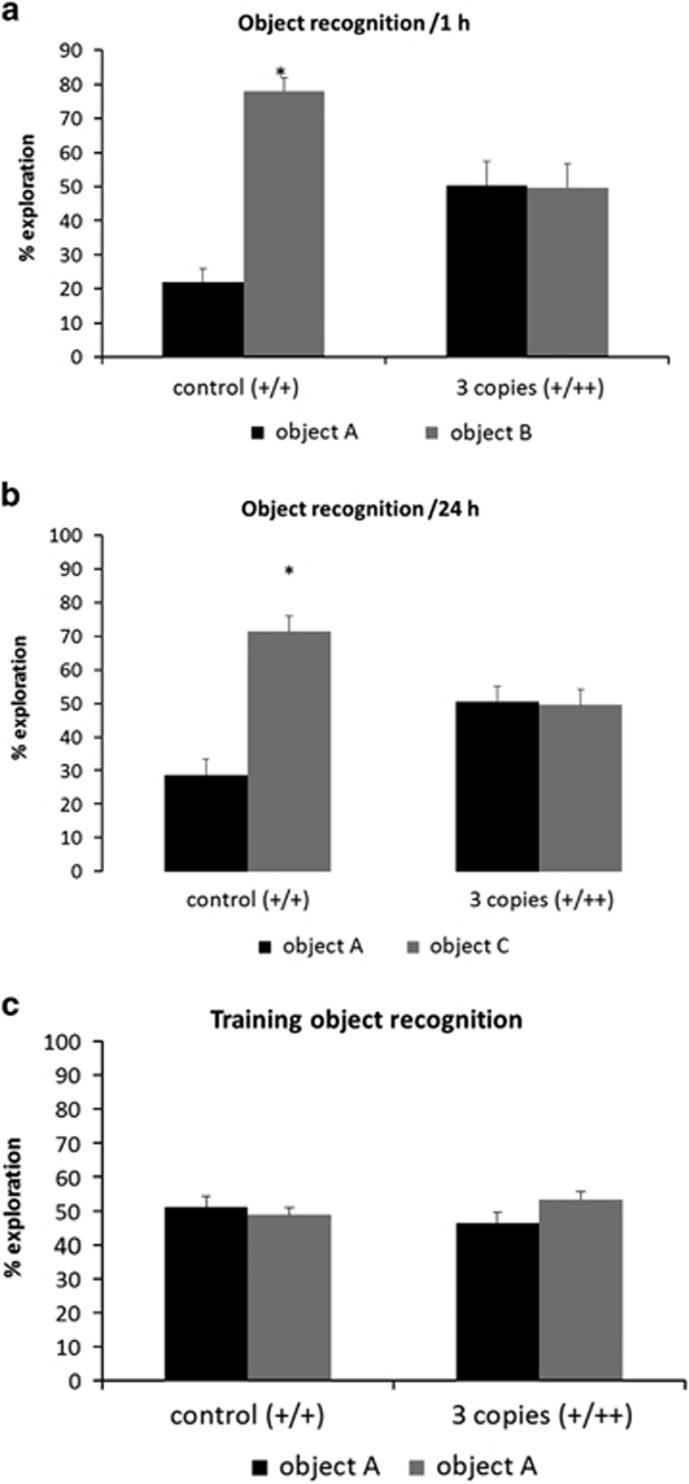
Novel object recognition test. Percentage of time spent exploring the familiar (object A) and the novel objects (object B and C) for control (+/+) and transgenic mice for the ACE gene (+/++).Object recognition after 1 (**a**) and 24 h (**b**) after training (**c**). As appropiate, repeated-measures analysis of variance followed by paired-sample *t*-test were performed. Data are reported as mean±s.e.m. *P<0.05 compared with the time exploring the familiar object.

For NOR, repeated-measures analysis of variance showed a significant effect of novel objects (within subject; F (1.22)=11.692; *P*<0.05) and an interaction between this factor and the genetic background (between factor; F (1.22)=12.166; *P*<0.05). Paired-samples *t*-test showed that transgenic mice did not preferentially explore the novel object in either sessions (at 1 and 24 h after familiarization), whereas control group spent more time exploring the novel object at 1 h (Figure 2a) and 24 h (Figure 2b) after the training session (*t* (11)=−7.152, −4.485; *P*<0.05, respectively). During the training session, with two identical objects, no significant difference in the time of exploration was observed (Figure 2c).

## Discussion

Our results suggest that ACE may have a role in cognitive deficits observed in patients with SCZ. First, we found higher ACE activity levels associated to a worse performance in verbal memory test for a sample comprising patients with SCZ and HCs. In parallel, we showed that mice with three copies of ACE genes displayed impairment in one cognitive test associated to short- and long-term memory. In addition, there was a significant difference in ACE activity and cognitive performance on several tests between SCZ patients and HCs.

Cognitive functioning is a core feature of SCZ. Approximately 80% of patients are clinically impaired in at least one cognitive domain.^55^ Cognitive performance was the best predictor of functional outcome for patients with SCZ.^56^ Moreover, cognitive decline starts before the onset of psychosis,^57^ suggesting that cognitive impairments might reflect the neurodysfunction process that underlies SCZ development rather than only disease progression processes.^58, 59^ Almost all cognitive functions are impaired at heterogeneous levels in different studies for each cognitive domain, in part, by methodological issues, but most probably also owing to true differences between the patients.^60^ In such context, verbal memory is among the most impaired cognitive domains in SCZ and unaffected siblings.^36, 61^ As expected, differences in cognitive performance between SCZ patients and HCs were observed for most tests used here.

Hopkins verbal learning test or Hopkins delayed recall is a three-trial list learning, and free recall task comprising 12 words, in which four words are from different semantic categories.^45^ The immediate recall can be considered a test for verbal learning or short-term memory, whereas the later free recall is a long-term verbal memory test, in which the information, after being out of consciousness flow need to be retrieved. In our sample, the association of ACE was stronger for later recall, suggesting an association to long-term memory.

The first issue to be considered is that ACE activity and Hopkins delayed recall measures were both highly different between SCZ patients and HCs (Figure 1). Therefore the significant correlation observed between them can reflect a statistical artifact. Nonetheless, the differences on cognitive tests performance of patients and HCs, considering Hopkins delayed recall (*t*=−5.585) as parameter, were higher for the Hopkins total measure (*t*=−6.422) and similar to the Letter–number task (*t*=−5.494). Thus, the results are not fully explained by the observed difference between patients and controls. Furthermore, there is a known previous specific association between ACE and memory.^24^ To better understand whether this was an effect of being a patient of having higher ACE levels, the sample was divided in groups based upon ACE activity levels, and we found that the Hopkins was significantly different between high- and low-activity groups, even correcting for case status (*P*=0.029). The scatter plots of Hopkins vs ACE measures suggest that the marked difference between patients and HC explain why the correlation is significant only with the whole sample, whereas the extreme group comparison reinforce a true association of ACE and memory, irrespective of clinical status. At this point, however, the results with humans must be considered with caution, because some did not survive considering the multiple comparison-adjusted *P*-value, which suggested not enough power to detect true effects. One additional limitation to the interpretation of the results is the use of antipsychotics in the patients group. Using a dose equivalence approach, the results seemed not to be significantly affected by antipsychotics, although we cannot rule out this possibility, as our controls were non-medicated.

The NOR test is based on the spontaneous tendency of rodents to spend more time exploring a novel object than a familiar one. The choice to explore the novel object reflects the use of learning and recognition memory.^49^ Transgenic animals with three copies showed a deficit in the recognition test performed at 1 and 24 h after the training session, indicating short- and long-term memory deficits (Figure 2). It has been previously described that ACE activity in these animals are increased to 144% compared with animals with two copies.^37^ This increase is not accompanied by changes in blood pressure.^37, 62^ Our animal model adds novelty to the current literature as it shows that differences in ACE availability can induce cognitive effects, which is in good agreement with the results on ACE and cognition in other neuropsychiatric diseases and different animal models. In fact, treatment with ACE inhibitors, specially using centrally active compounds (that is, perindopril or captopril), reduced the risk to develop both Alzheimer's disease and vascular dementia in patients,^27, 63, 64^ and this effect was significantly independent of the indication due to hypertension.^65^ In Alzheimer's disease animal model, perindopril, but not imidapril or enalapril (non-centrally active ACE inhibitors) reduced the cognitive impairment, independently of its antihypertensive effects.^66, 67^ Using the same NOR test, Yamada *et al.*^26^ found that perindopril improves the cognitive impairment in animal model of vascular dementia. Current literature on animals and humans suggest that the effects of ACE on cognition are independent of blood pressure variation, but as we did not directly measure the blood pressure of animals and humans in the current study, we cannot completely exclude this possibility, which need to be addressed in future replications.

The precise mechanisms underlying the impaired cognition observed for three-ACE copy mice are not completely clear at this point. However, one might consider the ACE roles in RAS, in which an increase in ACE activity could lead to an increase of Ang II which, in turn, was associated in several studies to impairments in learning and memory.^68, 69, 70^ The injection of Ang II in rat hippocampus inhibits the enhancement of long-term memory.^71^ In another study, the Ang II caused impairment in spatial memory, affecting the acquisition, consolidation and recall of memory, and significantly reduced acetylcholine levels without affecting the levels of acetylcholinesterase.^70^ Furthermore, the use of angiotensin type 1 blockers or ACE inhibitors were shown to improve memory and reverse cognitive deficits in animals.^67, 68^ However, as we did not measure the angiotensin compound levels, we cannot state that the observed results are only attributable to RAS. In fact, ACE is a ubiquitously expressed enzyme that can catalyze the cleavage of several other substrates known to impact the cognitive performance, such as the substance P^72^ and neurotensin.^73^ Furthermore, animal studies have shown that ACE inhibition improves memory function and prevents the impairment associated with cholinergic hypofunction, oxidative stress and the deposition of β-amyloid.^70, 74, 75, 76^ One additional possibility is through the association between ACE and dopaminergic neurotransmission. Indeed, acute and chronic cocaine administration induced a significant increase of ACE activity and gene expression in the rat prefrontal cortex and striatum, whereas ACE inhibitors showed a decrease in dopamine release.^5, 6^ Furthermore, both striatal and prefrontal function have been associated to performance on Hopkins delayed recall.^77^ Dopamine and glutamate systems manipulations can generate robust impairments in NOR.^52^ So, the dopamine-RAS^78^ interplay could explain ACE link to SCZ and cognitive deficits, representing it as a promising target to further investigation. Overall, the different described pathways can link ACE and cognition, but how this truly happens for SCZ needs to be further investigated.

Whenever investigating animal models, one major issue is whether the results of the tests can be considered a correct approximation to the human behavior and cognitive performance. At this point, the translational approach suggesting convergence to memory impairments with higher ACE activity levels reinforce our findings, although with some cautions. Otherwise, the National Institute of Mental Health Measurement and Treatment Research to Improve Cognition in Schizophrenia proposed both the Hopkins Verbal Learning—Immediate recall and NOR as part of consensual batteries, respectively in humans and animals, for the cognitive investigation in SCZ.^79, 80^ Although the tests are suggested to investigate SCZ, they do not measure exactly the same construct (verbal memory in humans vs recognition memory in animals) and there are differences in the timing of outcome measures (30 min for Hopkins delayed recall vs 24 h for NOR). So, there are some limitations on the translational approach adopted that prevent us to conclude, but not to suggest the association of ACE and memory in SCZ.

In conclusion, our study demonstrates a previously unreported association between ACE and cognitive measures in patients with SCZ. We show then that the modulation of ACE gene load is associated with cognitive performance in mice. Although previous studies have focused on Ang II, we provide here evidences that ACE can, by itself, contribute to cognitive phenotypes. These results are especially relevant considering that until now, there is no effective pharmacological treatment to cognitive deficits in SCZ. Future studies would clarify whether centrally active ACE inhibitors could be an add-on strategy on SCZ.

## Figures and Tables

**Table 1:** Sociodemographic characteristics

		*Patients (*n=*72)*		*Healthy controls (*n=*69)*		*Statistics*	
		N	*(%)*	N	*(%)*	*Test-value*	P*-value*
Gender	Male	50	(69.4)	44	(63.8)	0.511	0.475
	Female	22	30.6	25	31.2		
Educational level	Years	72	(11.11)	69	(11.33)	0.478	0.634
Age, mean	Years	72	(33.19)	69	(34.22)	−0.656	0.513

**Table 2:** Differences in tests performance between patients and healthy controls

*Cognitive domain*	*Test-value*	P*-value*
*Working memory/updating*		
Letter memory task	−1.784	0.0740
Keep track task	−4.117	<0.0001

*Inhibition*		
Stroop	−4.008	<0.0001

*Set-shifting tasks*		
Trail making test	−3.828	0.0002
Letter–number task	−5.494	<0.0001

*Verbal learning/memory*		
Hopkins total	−6.422	<0.0001
Hopkins delayed recall	−5.585	<0.0001

*Complex executive function*		
WCST-CAT	−1.600	0.1100

Abbreviation: WCST-CAT, Wisconsin card sorting task category score.

**Table 3:** Correlation between ACE enzymatic level and results of cognitive tests (adjusted for age, sex and IQ)

*Cognitive domain*	*Test-value*	P*-value*	*Test-value*	P*-value*
			*Corrected for clinical status*	
*Working memory/updating*				
Letter memory task	−0.035	0.682	0.026	0.767
Keep track task	−0.138	0.108	0.000	0.998

*Inhibition*				
Stroop	0.174	0.042	0.042	0.627

*Set-shifting tasks*				
Trail making test	0.010	0.906	−0.086	0.318
Letter–number task	0.118	0.169	−0.067	0.437

*Verbal learning/memory*				
Hopkins total	−0.218	0.010	−0.008	0.925
Hopkins evoc	−0.250	0.003	−0.076	0.377

*Complex executive function*				
WCST-CAT	0.021	0.803	−0.049	0.569

Abbreviations: ACE, angiotensin I-converting enzyme; WCST-CAT, Wisconsin card sorting task category score.
